# Comparison of T7 In Vitro Transcription and *E. coli* Expression Systems for RNAi-Based Control of *Euproctis pseudoconspersa* by Targeting *EpCHSA*

**DOI:** 10.3390/insects17050453

**Published:** 2026-04-24

**Authors:** Linyuan Huang, Fanhui Meng, Jinxiu Yu, Ying Luo, Zhen Liu, Wan Deng, Mi Li, Xiudan Wang, Yifei Xie

**Affiliations:** 1 Laboratory of Insect Behavior and Evolutionary Ecology, College of Life and Environmental Sciences, Central South University of Forestry and Technology, Changsha 410004, China; 17347701449@163.com (L.H.);; 2 Institute of Forestry and Grassland Protection, Hunan Academy of Forestry, Changsha 410004, China; 3 Yuelushan Laboratory, Changsha 410018, China; 4 Hunan Forest & Grassland Fire Prevention Center, Hunan Forestry Bureau, Changsha 410004, China; 5 College of Life Sciences, Hunan University of Arts and Science, Changde 415000, China

**Keywords:** *Euproctis pseudoconspersa*, chitin synthase A, dsRNA production, RNA interference

## Abstract

*Euproctis pseudoconspersa* is a devastating pest in *Camellia oleifera* plantations, necessitating the development of sustainable molecular intervention strategies. This study targeted the chitin synthase A gene (*EpCHSA*) to evaluate and compare the RNA interference (RNAi) efficacy of dsRNA synthesized via the T7 in vitro transcription system and the *Escherichia coli* HT115 (DE3) expression system. The *EpCHSA* gene (2199 bp ORF) was cloned and characterized, exhibiting peak expression during the fourth-instar stage, and predominantly in the head tissues of fifth-instar larvae. Bioassays demonstrated that larvae fed with 500 ng/μL in vitro synthesized dsRNA exhibited continuous gene silencing for five days, reaching a maximum efficiency of 68.1%. Conversely, treatment with 100× concentrated bacterial broth (5000 ng/μL) elicited a superior silencing effect of 79.3% within 24 h. Furthermore, the bacterial treatment group reached a 14-day mortality rate of 46.66%, significantly higher than the in vitro group (38.33%). Both methods induced severe phenotypic abnormalities, including molting failure and pupal malformation. These findings conclude that the *E. coli* expression system offers a cost-effective and highly potent platform for dsRNA production. This research provides a critical technical foundation and promising application prospects for the field-scale management of *E. pseudoconspersa* utilizing RNAi-based biopesticides.

## Simple Summary

This study compared T7 in vitro transcription and *E. coli* expression systems for dsRNA production against *Euproctis pseudoconspersa*. The bacterial system offered a cost-effective alternative, achieving superior *EpCHSA* silencing efficiency. This led to increased mortality and severe developmental deformities, providing a potent strategy for RNAi-based biopesticide development.

## 1. Introduction

As a woody oil crop endemic to China, *Camellia oleifera* holds immense economic value, with an industry output surpassing 100 billion RMB in 2020 that significantly contributes to rural agricultural economies. However, the continuous expansion of cultivation areas has increasingly intensified challenges related to pest and disease management [1,2]. The oil-tea tussock moth, *Euproctis pseudoconspersa* (Lepidoptera: Lymantriinae), is one of the most destructive pests in China’s major oil-tea production regions. Characterized by significant gregariousness and voracious feeding behavior, its larvae directly diminish yields and degrade oil quality by consuming foliage and fruit, resulting in substantial economic losses [3]. Current management strategies rely primarily on physical removal, chemical pesticides, and conventional biological control. Nevertheless, physical methods often suffer from low efficiency, while chemical pesticides pose risks of environmental contamination and food safety concerns; furthermore, traditional biological control is frequently constrained by climatic and regional factors. Consequently, there is an urgent necessity to develop novel, highly efficient, and environmentally benign control technologies.

RNA interference (RNAi) technology, which induces the degradation of target mRNA through the delivery of specific double-stranded RNA (dsRNA), has emerged as a research hotspot in green pest management due to its high specificity, environmental compatibility, and low risk of resistance development [4,5]. Chitin serves as a critical structural component of the insect exoskeleton and peritrophic matrix, with its biosynthesis catalyzed by chitin synthase A (CHSA) [6,7]. Given that the chitin biosynthetic pathway is absent in higher plants and vertebrates, and that CHSA is primarily responsible for chitin deposition in the cuticle and tracheae, this enzyme represents an ideal molecular target for RNAi-based interventions [8,9]. Although the oral delivery of dsRNA offers advantages such as operational simplicity and scalability, the practical implementation of RNAi in Lepidoptera remains constrained by significant bottlenecks, including the rapid degradation of dsRNA, low delivery efficiency, and high production costs [10,11,12,13,14,15].

Currently, the synthesis of double-stranded RNA (dsRNA) is primarily achieved through two methods: in vitro chemical transcription and in vivo biosynthesis. While the former is constrained by high costs that limit its large-scale application, in vivo expression using the RNase III-deficient *Escherichia coli* strain HT115(DE3) demonstrates broader application prospects due to its cost-effectiveness, high yield, and scalability [16]. However, a systematic evaluation regarding the comparative RNA interference (RNAi) efficiency of these two synthesis methods in the oil-tea tussock moth, *Euproctis pseudoconspersa*, remains lacking. In this study, we first cloned the chitin synthase A gene (*EpCHSA*) from *E. pseudoconspersa* and elucidated its spatiotemporal expression patterns. Subsequently, we comparatively evaluated the differences in gene silencing efficiency, lethal effects, and developmental regulation between dsRNA produced via the T7 in vitro transcription system and via the L4440-HT115 bacterial expression system. This work aims to provide molecular targets and technical support for the precise, green management of *E. pseudoconspersa*.

## 2. Materials and Methods

### 2.1. Insects

Specimens of the oil-tea tussock moth, *E. pseudoconspersa*, were collected from *C. oleifera* plantations in Huaihua City, Hunan Province, China. The population was maintained in the laboratory within a climate-controlled chamber under the following conditions: a temperature of 25 ± 1 °C, 75–80% relative humidity (RH), and a photoperiod of 15:9 (L:D) h. The larvae were reared on a diet of fresh *C. oleifera* foliage.

### 2.2. RNA Extraction and cDNA Synthesis

Total RNA was extracted from samples representing different developmental stages (eggs, 1st–5th-instar larvae, male and female pupae, and male and female adults) and from specific tissues dissected from 5th-instar larvae (head, thorax, midgut, epidermis, and fat body) using the Fast Total RNA Kit (Baoguang Biotech Co., Ltd., Chongqing, China). The purified RNA was aliquoted into labeled nuclease-free microcentrifuge tubes and stored at −80 °C for subsequent analysis. All experiments were performed with three biological replicates. First-strand cDNA was synthesized using the ExonScript RT Kit (Baoguang Biotech Co., Ltd., Chongqing, China) according to the manufacturer’s instructions.

### 2.3. Cloning and Bioinformatics Analysis of the EpCHSA Gene

Total RNA was isolated from 4th-instar larvae and reverse-transcribed into cDNA. Specific primers were designed based on conserved CHSA gene sequences from closely related species Table S1). The sequence of the *EpCHSA* gene was obtained via PCR amplification, followed by molecular cloning and sequencing. The transmembrane domains of the protein were predicted using the TMHMM Server v.2.0, and the open reading frame (ORF) was identified using the NCBI ORF Finder (National Library of Medicine (NLM), Bethesda, MD, USA). For phylogenetic analysis, homologous CHSA sequences from selected Lepidopteran species were retrieved from the NCBI GenBank database (Table S2). Multiple sequence alignments were performed using DNAMAN 9.0, and a phylogenetic tree was constructed using MEGA 11.

### 2.4. Analysis of Spatiotemporal Expression Patterns

The spatiotemporal expression profiles of *EpCHSA* were determined using quantitative real-time PCR (RT-qPCR). Samples were collected from various developmental stages (eggs, 1st–5th-instar larvae, male/female pupae, and male/female adults) and specific tissues dissected from 5th-instar larvae (head, thorax, midgut, epidermis, and fat body). Three biological replicates were performed for each sample, with β-actin serving as the internal reference gene. The specific primer sequences are listed in Supplementary Table S1.

The qPCR reaction was conducted in a 20 μL total volume containing 10 μL of 2× SP qPCR Mix, 0.5 μL each of the forward and reverse primers, 1 μL of cDNA template, and nuclease-free water to adjust the final volume. The thermal cycling conditions were as follows: initial denaturation at 94 °C for 20 s, followed by 40 cycles of denaturation at 94 °C for 10 s, annealing at 60 °C for 10 s, and extension at 72 °C for 10 s.

### 2.5. Preparation of dsRNA

Synthesis via In Vitro Transcription (T7 System, Promega Corporation, Madison, WI, USA): Primers containing the T7 promoter sequence were designed to amplify the *EpCHSA* target fragment (yielding a 1060 bp product) and the control EGFP fragment yielding a 436 bp product) (Supplementary Table S1). dsRNA was synthesized using the T7 RiboMAX^TM^ Express RNAi System (Promega Corporation, Madison, WI, USA). The 20 μL reaction mixture consisted of 10 μL of 2× Reaction Buffer, 1 μg of DNA template (5 μL), 2 μL of T7 Enzyme Mix, and nuclease-free water to adjust the final volume. The mixture was incubated at 37 °C for 2 h. To promote annealing of the complementary strands, the reaction was heated to 70 °C for 10 min and subsequently allowed to cool to room temperature. The synthesized dsRNA was purified via ethanol precipitation. Finally, the concentration and purity of the dsRNA were assessed using a spectrophotometer (applying the standard RNA conversion factor of 1 OD_260_ = 40 μg/mL).

Production via Bacterial Expression (HT115): The *EpCHSA* fragment was cloned into the L4440 vector and transformed into *E. coli* DH5α competent cells. Following overnight culture, single colonies were selected, and the plasmid insertion was verified by double digestion with SacI and HindIII. The verified recombinant plasmid was then transformed into the RNase III-deficient *E. coli* strain HT115(DE3) to generate the engineered strain *EpCHSA*-L4440-HT115. To induce dsRNA expression, the bacteria were cultured at 37 °C until the optical density at 600 nm (OD_600_) reached 0.4. Isopropyl β-D-1-thiogalactopyranoside (IPTG) was added to a final concentration of 0.4 mM, and the culture was incubated for an additional 4.5 h. Bacterial cells were harvested by centrifugation and resuspended in 1 mL of sterile water to produce the bacterial dsRNA formulation. For the bacteria-mediated dsRNA formulations, the absolute dsRNA yield was first determined via extraction. Specifically, total nucleic acids were extracted from a 1 mL aliquot of the IPTG-induced culture using a Fast Total RNA Kit according to the manufacturer’s protocol. The extracted nucleic acids were subsequently treated with RNase A and DNase I to eliminate single-stranded RNA and DNA. The purified dsRNA was then prepared for agarose gel electrophoresis visualization and quantified using a spectrophotometer, establishing a baseline yield of approximately 50 ng/μL for the original culture (designated as the 1× formulation). Based on this baseline quantification, higher-concentration bacterial formulations (10× and 100×) were prepared utilizing strict volumetric concentration ratios.

To ensure a rigorous and accurate comparison between the two delivery systems, the exact same target fragment of the *EpCHSA* gene was utilized for both the in vitro transcription and the construction of the bacterial expression vector. For in vitro transcription synthesis, this specific fragment was amplified using primers containing T7 promoters. Concurrently, the identical target sequence was cloned into the L4440 vector for expression in *E. coli* HT115. Therefore, the core active sequence and length of the dsRNA produced by both systems are completely identical.

### 2.6. RNAi Silencing Efficacy Determination

Third-instar larvae were selected and subjected to starvation for 12 h prior to experimentation. Each dsRNA formulation was uniformly brushed onto the adaxial surface of clean *Camellia oleifera* leaves at a precise ratio of 100 μL per 1 g of leaf mass. After air-drying for 30 min to ensure complete absorption, the treated leaves (alongside identically prepared water and empty-vector controls) were fed to starved larvae. The larvae were then fed *C. oleifera* leaves coated with one of two types of dsRNA preparations: (1) dsRNA synthesized via the T7 kit at concentrations of 100, 500, and 1000 ng/μL; or (2) bacterial formulations expressing dsRNA at concentrations of 1×, 10×, and 100×. Leaves treated with synthesized ds*EGFP* (Enhanced Green Fluorescent Protein) and ds*EGFP*-expressing bacteria (L4440-HT115) served as negative controls for the in vitro synthesis and bacterial expression groups, respectively.

Treated leaves were replaced every 24 h. To evaluate silencing effects, samples were collected on days 1, 3, and 5 post-treatment, and the relative expression levels of *EpCHSA* were quantified via RT-qPCR. Each treatment group consisted of 40 larvae, and all experiments were performed in biological triplicate.

### 2.7. Effects of dsRNA on Survival and Development

Survival and Molting Assay (3rd-Instar Larvae): To investigate the comparative effects of the two dsRNA delivery methods on the survival of *Euproctis pseudoconspersa*, 3rd-instar larvae were randomly allocated into four groups (n = 60 per group) and subjected to differential feeding treatments: (1) dsEGFP (control for in vitro synthesis); (2) 500 ng/μL synthesized dsEpCHSA; (3) dsEGFP-HT115 (control for bacterial expression); (4) 10× (500 ng/μL) dsEpCHSA-HT115 bacteria. The bioassay spanned a 14-day period. Larvae were provided with dsRNA-treated leaves on Day 1 and Day 7; during the intervening intervals, they were fed fresh leaves treated with sterile water. Survival rates and molting progress were recorded daily at a fixed time.

Pupation and Gene Expression Assay (5th-Instar Larvae): Fifth-instar larvae were treated with leaves coated with the 100× dsEpCHSA-HT115 bacterial formulation, with the dsEGFP-HT115 group serving as the control. The preparation of dsRNA-treated leaves was performed as described in Section 2.6. Treated leaves were replaced every 24 h. Following two consecutive days of treatment, the larvae were switched to a diet of untreated leaves. Pupal morphology was monitored daily until pupation was complete for the entire cohort. Upon the onset of pupation, 9 live individuals on the first day of the pupal stage were sampled from both the experimental and control groups to quantify the relative expression of the target gene. All assays were performed with three biological replicates.

### 2.8. Statistical Analysis

Data processing and graph generation were performed using GraphPad Prism 5.0 software. Quantitative PCR (qPCR) data are presented as the mean ± standard error of the mean (SEM). The relative expression level was calculated using the 2^−ΔΔCt^ method [17]. For the spatiotemporal expression patterns and effects of RNAi on pupation, statistical significance was determined using a one-way analysis of variance (ANOVA) followed by Tukey’s multiple comparison test. For the 14-day bioassay, insect survival data were analyzed using the Kaplan–Meier method, and statistical differences between the survival curves of the different dsRNA treatment groups were determined using the Log-rank (Mantel–Cox) test. Differences were considered statistically significant at *p* < 0.05.

## 3. Results

### 3.1. Characterization of EpCHSA

A partial sequence of the *EpCHSA* gene was successfully cloned, containing an open reading frame (ORF) that encodes 733 amino acids. Basic bioinformatic analysis indicated that this fragment encompasses the essential Glycosyltransferase Family 2 domain and nine putative transmembrane segments (Figure S1A).

Furthermore, a phylogenetic tree constructed using the Neighbor-Joining (NJ) method demonstrated that *EpCHSA* is clustered most closely with the *CHS* genes of *H. cunea* and *Mythimna separata* (Figure S1B).

### 3.2. Spatiotemporal Expression Patterns of EpCHSA

**Figure 1:**
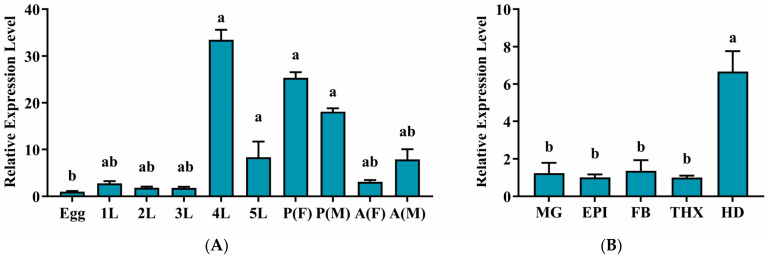
Relative expression levels of the *EpCHSA* gene in *Euproctis pseudoconspersa*. (**A**) Expression patterns across different developmental stages. Abbreviations: Egg; 1L–5L: 1st- to 5th-instar larvae; P: pupae; A: adults; F: female; M: Male. (**B**) Tissue-specific expression in 5th instar larvae. Abbreviations: MG: midgut; EPI: epidermis; FB: fat body; THX: thorax; HD: head. Statistical Analysis: Data were analyzed using one-way ANOVA. Different letters (a, b, ab) above the bars indicate statistically significant differences (*p* < 0.05).

The relative expression levels of *EpCHSA* across different developmental stages were determined via RT-qPCR. The results indicated that *EpCHSA* was expressed constitutively throughout all developmental stages of *E. pseudoconspersa*. Expression peaked at the 4th-instar larval stage, reaching levels approximately 46.4 times that of those observed in the egg stage, where expression was lowest. No significant differences were observed among the other developmental stages (*p* > 0.05). Furthermore, no sexual dimorphism in expression levels was detected in either the pupal or adult stages (Figure 1A).

Tissue-specific expression analysis in 5th-instar larvae revealed that *EpCHSA* transcript levels were significantly higher in the head compared to all other tested tissues (*p* < 0.05). Specifically, expression in the head was 3.46, 4.23, 3.11, and 4.27 times that in the midgut, epidermis, fat body, and thorax, respectively. No significant differences were found among the non-cephalic tissues (Figure 1B). These high expression levels strongly indicate that *EpCHSA* plays a critically essential role in the intensive feeding and molting processes during the 4th-instar developmental stage, as well as in head-specific physiological functions.

### 3.3. Silencing Efficacy of EpCHSA

Prior to conducting the bioassays, the integrity and correct molecular size of the dsRNA synthesized via both the in vitro transcription system and the *E. coli* HT115 bacterial expression system were verified by agarose gel electrophoresis (Figure S1C). As expected, the in vitro transcribed dsRNA exhibited a single, highly pure band, whereas the bacterially derived formulation displayed the distinct target dsRNA band alongside typical endogenous bacterial RNAs (e.g., rRNA and tRNA).

**Figure 2:**
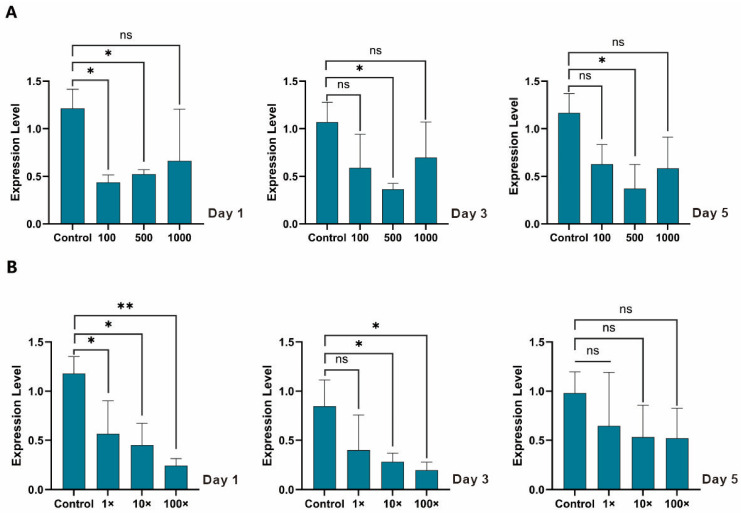
RNAi silencing effects of *EpCHSA* in *Euproctis pseudoconspersa*. (**A**) Efficacy of dsRNA synthesized via the T7 in vitro transcription system. (**B**) Efficacy of bacterially expressed dsRNA produced in *Escherichia coli*. Data were analyzed using *t*-test. Asterisks indicate statistically significant differences compared to the corresponding control group (* *p* < 0.05; ** *p* < 0.01; ns not significant).

To determine the optimal concentration for RNA interference, larvae were treated with in vitro synthesized dsEpCHSA at concentrations of 100, 500, and 1000 ng/μL. The results indicated that 500 ng/μL was the most effective concentration. At this dosage, *EpCHSA* transcript levels decreased by 58.3%, 65.8%, and 68.1% on days 1, 3, and 5 post-treatment, respectively. These reductions were statistically significant compared to the dsEGFP control group (*p* < 0.05). In contrast, the 100 ng/μL treatment group showed a significant reduction of 64.1% only on day 1 (Figure 2A), with no significant differences observed on days 3 and 5 (Figure 2B).

The silencing effects of dsRNA-expressing bacteria were evaluated at three concentrations: 1× (50 ng/μL), 10× (500 ng/μL), and 100× (5000 ng/μL). On day 1, the 100× treatment group exhibited a highly significant reduction in *EpCHSA* expression of 79.3% (*p* < 0.01). The 10× and 1× groups also showed significant reductions of 61.8% and 52.1%, respectively (*p* < 0.05) (Figure 2B). By day 3, significant suppression persisted in the 100× and 10× groups, whereas no significant difference was observed in the 1× group (Figure 2B). By day 5, no statistically significant differences in gene expression were detected among any of the treatment groups (Figure 2B).

### 3.4. Effects of RNAi on Survival and Molting

Based on the optimal and sustained gene silencing efficiencies observed in the preceding dose–response assays (Section 3.3), a dsRNA concentration of 500 ng/μL (and its equivalent 10× bacterial formulation) was rationally selected for the 14-day survival bioassays. This unified concentration also ensured a fair, head-to-head comparative analysis between the two distinct delivery systems.

**Figure 3:**
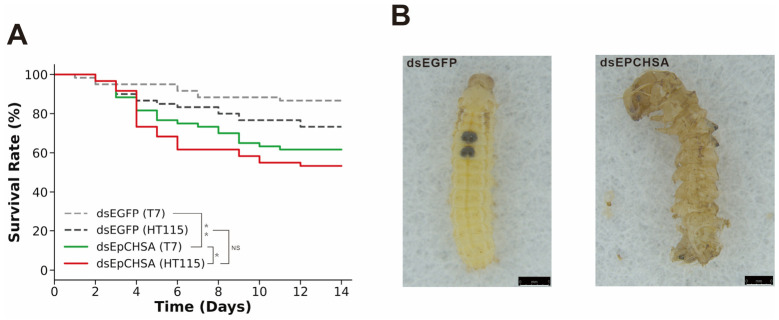
Effects of RNAi-mediated *EpCHSA* silencing on the survival and pupation of *Euproctis pseudoconspersa*. (**A**) Analysis of cumulative larval mortality rates following dsRNA treatment. Survival curves were generated using the Kaplan–Meier method, and statistical significance between groups was evaluated using the Log-rank (Mantel–Cox) test. Asterisks indicate significant differences (* *p* < 0.05; ** *p* < 0.01; NS: not significant). (**B**) Phenotypic malformations observed during pupation. Asterisks indicate statistically significant differences compared to the control group.

Larval survival was monitored continuously over a 14-day post-feeding period. By the end of the assay, the raw cumulative mortality rates for the dsEGFP and dsEGFP-HT115 control groups were 18% and 26.7%, respectively. Importantly, a direct pairwise comparison between these two control groups revealed no significant difference (Log-rank test, *p* > 0.05), confirming that the bacterial vector alone did not induce substantial toxicity. In contrast, treatments with the T7-synthesized dsEpCHSA and HT115-expressed dsEpCHSA resulted in considerably higher raw mortality rates of 38.3% and 46.6%, respectively. Kaplan–Meier survival analysis followed by the Log-rank test demonstrated that the dsEpCHSA treatments significantly reduced larval survival compared to their respective dsEGFP controls in both the T7 system (*p* = 0.002) and the HT115 system (*p* = 0.025), thereby validating the robust insecticidal efficacy of targeting *EpCHSA* (Figure 3A).

Furthermore, pairwise comparisons revealed no significant difference in the overall survival distributions between larvae treated with the HT115-expressed ds*EpCHSA* and those treated with the T7-synthesized ds*EpCHSA* (Log-rank test, *p* = 0.367). This indicates that the bacterial delivery system provides comparable pest control efficacy to the conventional in vitro synthesis method.

As shown in Figure 3B, compared to the normal development observed in the control group, larvae in both the T7-synthesized and HT115-expressed dsEpCHSA treatment groups exhibited severe phenotypic defects during the transition to the 4th instar. These defects included melanization of the body wall and the failure to shed the old cuticle (ecdysis failure). Although the exact frequency of each morphological defect was not independently quantified, daily observations indicated that these ecdysis failures were the predominant lethal phenotypes directly responsible for the high mortality rates recorded in the treatment groups.

### 3.5. Effects of RNAi on Pupation

**Figure 4:**
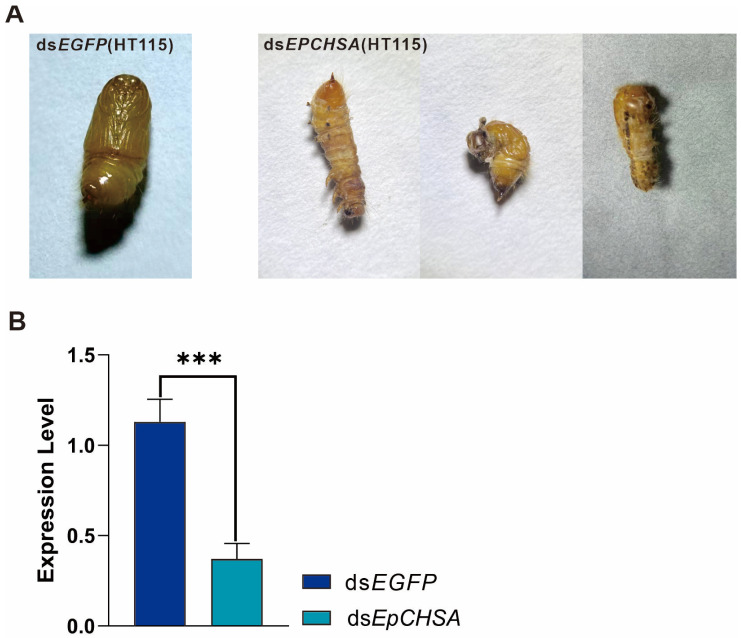
Impact of RNAi-mediated *EpCHSA* silencing on pupation. (**A**) Representative phenotypes of pupal malformations observed in the treatment groups. (**B**) Relative expression levels of *EpCHSA* in the pupal stage following dsRNA treatment. Triple asterisks indicate statistically significant differences compared to the control group (*** *p* < 0.001).

Phenotypic variations during pupation were monitored and recorded across the different treatment groups. The observed malformations in the treated insects were categorized into three distinct phenotypes: (1) Type I: Characterized by a curled pupal body and abdominal torsion; (2) Type II: Characterized by the failure of the 8th and 9th abdominal segments to differentiate, retaining larval characteristics; (3) Type III: Characterized by indistinct boundaries between the head and thorax. While the region posterior to the thorax pupated successfully, the anterior region retained a larval-like state (Figure 4A).

To validate the RNAi efficacy at the molecular level, gene expression in the pupal stage was assessed via RT-qPCR. The results demonstrated a 55.8% downregulation of *EpCHSA* transcripts in the treatment group compared to the control. This reduction was statistically highly significant (*p* < 0.001) (Figure 4B).

## 4. Discussion

In this study, the successful characterization of the *EpCHSA* fragment and the subsequent evaluation of two distinct dsRNA delivery systems demonstrated the robust potential of RNAi for controlling *E. pseudoconspersa*. Prior to conducting RNAi bioassays, it is crucial to validate the identity and functional relevance of the molecular target. Sequence analysis revealed that our cloned 733-aa fragment of *EpCHSA* represents a highly conserved partial sequence. Alignment with the full-length *CHSA* sequence of the related Lepidopteran *Hyphantria cunea* (1565 amino acids) indicates that this fragment covers the central-to-C-terminal region, specifically corresponding to residues 833 to 1565. Conserved domain prediction revealed that this fragment possesses the essential hallmark motifs required for accurate functional classification. Specifically, the N-terminal portion encompasses the highly conserved Glycosyltransferase Family 2 domain (Glyco_transf_2, PF03160), representing the core catalytic domain of chitin synthases. Additionally, the C-terminal portion is highly hydrophobic and contains nine putative transmembrane segments. These TM domains anchor chitin synthase to the cell membrane, enabling extracellular chitin extrusion. This structural feature perfectly explains our RNAi outcomes: silencing this membrane-bound enzyme disrupts surface cuticular assembly, causing severe ecdysis failures and malformations. Furthermore, a phylogenetic tree constructed using the Neighbor-Joining (NJ) method demonstrated that *EpCHSA* is clustered most closely with the CHS genes of *H. cunea* and *M. separata* (Figure S1B). Together, these structural and evolutionary features unequivocally confirm the identity of the cloned fragment as a functional region of *EpCHSA*, thus providing an optimal and highly specific target pool for effective siRNA generation.

Building upon this robust target validation, we systematically evaluated the comparative efficacy of two dsRNA delivery methods—in vitro transcription (using the T7 RiboMAX^TM^ Express RNAi System) and bacterial expression—for RNAi-based management of *E. pseudoconspersa*. By employing the established inducible expression system in engineered bacteria, we ensured the efficient and stable production of dsRNA. The results demonstrated that both delivery strategies effectively silenced the *EpCHSA* gene. Treated larvae exhibited significant phenotypes, including developmental retardation, aberrant molting, and pupation failure. Among the highly susceptible individuals within our treatment groups, mortality occurred within 48–72 h, with cumulative mortality rates exceeding 45%. These observations from our current study are consistent with those of Miao et al. [18], who utilized dietary dsRNA delivery to target the *CHSA* gene in the green peach aphid (*Myzus persicae*). Their study reported that silencing *MpCHSA* disrupted cuticle synthesis, resulting in molting failure in 41.7% of nymphs and a significant reduction in survival rates. Similarly, Du et al. [19] employed both microinjection and feeding methods to silence the *CHSB* gene in the rusty grain beetle (*Cryptolestes ferrugineus*), observing phenotypes such as body curling, abdominal shriveling, and impaired locomotion in larvae. Furthermore, Zhang et al. introduced *LmCHSA*-targeting dsRNA into *Locusta migratoria manilensis* larvae via injection, which induced developmental retardation, molting defects, and morphological malformations [20]. Comparable lethal phenotypes have also been documented following the silencing of *CHSA* or *CHSB* genes in other species, including *Cnaphalocrocis medinalis*, *Henosepilachna vigintioctopunctata*, and *Blattella germanica* [21,22,23,24,25,26]. Collectively, these studies indicate that silencing *CHSA* and its orthologs disrupts the insect chitin biosynthesis pathway. This disruption triggers characteristic phenotypes—including aberrant molting, developmental stagnation, and cuticular malformations—ultimately leading to arrested development and significant mortality.

Although our expression profiling revealed that *EpCHSA* peaked in 4th-instar larvae and head tissues, our RNAi bioassays were strategically executed using 3rd-instar larvae. The rationale for this design is twofold. First, high transcript abundance indicates a peak physiological requirement for *CHSA* during the 4th-instar molting phase. By administering dsRNA during the preceding 3rd-instar stage, our objective was to pre-emptively exhaust the target transcript pool before the larvae reached this peak requirement, which effectively disrupted their subsequent molting and development. Second, from an agricultural perspective, deploying pest control interventions at earlier developmental stages (such as the 3rd instar) is far more critical and practical for preventing massive foliar damage in *Camellia oleifera* plantations. Therefore, the spatiotemporal expression profile served as a developmental functional guide to identify the gene’s most critical period, rather than a direct indicator of optimal RNAi sensitivity.

The dsRNA synthesized via the T7 in vitro transcription system exhibited optimal silencing efficacy at a concentration of 500 ng/µL. At this dosage, significant gene silencing was not only maintained throughout the experimental period but also showed no obvious temporal attenuation. In contrast, while the 100 ng/µL treatment induced significant silencing on day 1, its efficacy gradually attenuated over time, as evidenced by the gradual recovery of transcript levels and the loss of statistical significance on subsequent observation days (Days 3 and 5 post-treatment, Figure 2A). This temporal attenuation indicates that this lower dose is insufficient to maintain the RNAi machinery against continuous target gene transcription. Interestingly, the highest concentration (1000 ng/µL) showed a moderate inhibitory trend (30–50%) but failed to achieve statistical significance, indicating a non-linear relationship between dosage and temporal efficacy [27]. We hypothesize that excessive concentrations of exogenous dsRNA may activate pattern recognition receptors (such as the Toll or IMD pathways) within the insect, triggering non-specific immune responses that subsequently degrade the foreign nucleic acids or interfere with core components of the RNAi machinery. This hypothesis is indirectly supported by the existing literature demonstrating that exogenous dsRNA can function as a viral pathogen-associated molecular pattern (PAMP) in insects. For example, Prince et al. [28] demonstrated that dsRNA sensing elicits broad antiviral responses in insects, actively engaging the Toll signaling pathway. Furthermore, extensive functional crosstalk between the Toll and IMD pathways has been well-documented in various insect species [29]. These collective findings suggest that an excessive intracellular accumulation of dsRNA may overstimulate these interconnected immune networks, thereby triggering non-specific nuclease degradation or competitively saturating the core components of the RNAi machinery. Alternatively, in the complex in vivo environment, high-concentration dsRNA might be more prone to forming aggregates or being rapidly recognized and cleared by nucleases, thereby reducing the actual effective intracellular concentration. This phenomenon of “high-dose inefficacy” is highly consistent with the findings of Yang et al. [30], who observed a similar inhibition failure at 1000 ng/µL during RNAi against GSTs2 in *Spodoptera frugiperda*. Furthermore, Jian et al. [31] noted that gene silencing efficacy is modulated by dsRNA exposure concentration, duration, and species-specific variations in RNAi pathways, suggesting that overly high concentrations may lead to saturation of the RNAi machinery or the activation of non-specific immune defenses. These findings highlight the critical necessity of dose gradient optimization in practical applications to circumvent the triggering of insect defense mechanisms [32]. In future RNAi research, establishing precise dose–response curves correlating dsRNA intake with the degree of gene silencing will be essential [33]. Selecting a concentration that ensures robust silencing without inducing off-target effects or immune saturation will facilitate the transition of RNAi from qualitative observation to quantitative precision. Given the variations in RNAi susceptibility among different host insects and target genes, optimizing dsRNA dosages on a case-by-case basis is of paramount importance for the accurate and effective application of RNAi-based biopesticides in pest management.

Bacteria-mediated RNAi (BM-RNAi) offers a highly versatile and cost-effective platform for large-scale dsRNA production. Previous studies have shown that intact bacterial cell walls effectively shield dsRNA from rapid degradation by gut nucleases and alkaline environments [32]. In the present study, this protective encapsulation facilitated the sustained, localized release of dsRNA within the gut lumen. This generated a high-concentration microenvironment that triggered a robust initial downregulation of *EpCHSA* (79.3% by day 1 at the 100× concentration) and subsequent molting failure. However, the silencing effect diminished significantly after day 5. This attenuation suggests that prolonged dsRNA exposure and bacterial accumulation may trigger the insect’s innate immune response, thereby accelerating endogenous RNA clearance [34,35]. Beyond conventional dsRNases, this rapid turnover might also be mediated by the upregulation of specific host nucleases, akin to the *eri-1* gene in *Caenorhabditis elegans* [36] or gut-specific RNases in *Ostrinia furnacalis* [37]. To counteract this attenuation, we employed a repeated-feeding strategy. This approach effectively reactivated the RNAi machinery—likely by replenishing the dsRNA pool to sustain the activity of RISC (RNA-induced silencing complex)-associated proteins, such as the Argonaute family. Consequently, the cumulative mortality rate was elevated to over 45%, consistent with previous findings in *Spodoptera frugiperda* [38]. Moving forward, integrating BM-RNAi with advanced delivery strategies could further optimize its field efficacy. For instance, utilizing heat-inactivated engineered bacteria can preserve dsRNA integrity while minimizing the host’s antibacterial immune response [39]. Furthermore, conjugating this system with nanocarriers to establish a “dual-protection” platform, combined with optimized feeding regimens, warrants rigorous validation for practical pest management applications.

In the survival and developmental bioassays, the engineered bacteria treatment exhibited a higher mortality rate compared to the in vitro synthesis group. This outcome strongly aligns with previous findings; for instance, Ganbaatar et al. reported that feeding *Mythimna separata* larvae with inactivated *E. coli* expressing chitinase increased mortality by 10.8% and reduced body weight by 33.3% at 7 days post-treatment [40]. Similarly, Israni et al. demonstrated that feeding dsRNA-expressing live bacteria to *Plutella xylostella* and *Helicoverpa armigera* severely hindered larval molting and significantly suppressed adult fecundity and egg hatchability [41]. These results corroborate the potent lethality observed in our bacterial group, further validating the potential of bacteria-mediated dsRNA delivery in pest management. The sustained efficacy of the high-concentration bacterial formulation (e.g., 100×, equivalent to 5000 ng/µL), which notably circumvented the ‘high-dose inefficacy’ observed with 1000 ng/µL naked dsRNA, can likely be attributed to altered release kinetics. Unlike naked dsRNA, which causes a sudden luminal influx that may saturate the cellular RNAi machinery or trigger immediate immune clearance, bacterial cells act as a natural slow-release delivery system [42]. Prior to ingestion, the aqueous bacterial suspension maintains the cells in a non-proliferative state with structurally intact cell envelopes [43]. Crucially, the RNase III deficiency of the HT115 strain protects the encapsulated dsRNA from endogenous degradation during this starvation phase, ensuring formulation stability [16,44]. Upon ingestion, the bacterial cell wall robustly shields the encapsulated dsRNA from rapid degradation by insect midgut nucleases during transit [45]. Rather than surviving indefinitely in the hostile digestive juices, the bacteria are gradually lysed by midgut enzymes. This gradual lysis facilitates a steady, localized release of dsRNA directly in the vicinity of the midgut epithelial microvilli, followed by efficient cellular uptake primarily via clathrin-mediated endocytosis [46]. This protective and controlled-release mechanism ensures prolonged intracellular bioavailability without inducing immediate immune overload. In contrast, dsRNA synthesized via the T7 RiboMAX^TM^ Express system exists as naked molecules. Supported by our RT-qPCR results (Figure 2), the in vitro synthesized dsRNA maintained a continuous silencing effect lasting up to 5 days. While highly pure, naked dsRNA is indeed intrinsically vulnerable to rapid degradation by environmental and luminal nucleases [47], the optimal initial dosage utilized in our study (500 ng/µL) likely compensated for this extracellular degradation threshold. This ensured that a sufficient quantity of dsRNA was efficiently internalized to activate the intracellular RNAi machinery. Once internalized, the highly stable RISC drives the sustained gene suppression over the 5-day period [48]. Consequently, this high-purity formulation remains highly reliable and ideal for precise functional genomic assays under controlled laboratory conditions. While the cost-effective bacterial system holds immense promise for large-scale production, future research should focus on optimizing vector construction to enhance targeting specificity and lethality against *E. pseudoconspersa*. Moving forward, integrating this bacteria-mediated RNAi technology with conventional biological and chemical controls to formulate a comprehensive Integrated Pest Management strategy, alongside long-term field trials to evaluate ecological biosafety, will be crucial for its practical agricultural application.

A major bottleneck for the commercial application of RNAi biopesticides is the prohibitive cost and limited scalability of conventional in vitro transcription. To substantiate the economic viability of our *E. coli* HT115 system, we conducted a quantitative cost–yield analysis. Our bacterial platform achieved a robust yield of 140–150 mg of dsRNA per liter of culture. Factoring in the routine expenses for culture media, IPTG induction, and RNA extraction, the average production cost was merely 0.33–0.96 RMB per milligram. In stark contrast, the cost of commercial in vitro transcription synthesis—including templates, reagents, and purification—surged to 263–550 RMB per milligram. By reducing production costs by several hundred-fold while ensuring high yields, our quantitative metrics robustly demonstrate that this bacterial expression system is a highly cost-effective and scalable strategy. This aligns with the growing consensus that microbial production is crucial for the field-level viability of RNAi technology [49,50,51].

Building upon this economic reality, it is important to note that the concentrations of dsRNA used in our leaf-coating assays differed between the two systems (500 ng/µL for T7 system and 5000 ng/µL for the bacterial system). A direct comparison of molecular silencing efficiency would strictly require equimolar dosages, ideally delivered via microinjection. However, the primary objective of this study was to evaluate the practical field-application efficacy of these delivery methods. Given the prohibitive cost of in vitro transcription synthesis discussed above, applying high concentrations (e.g., 5000 ng/µL) is economically unfeasible for pest management. Conversely, the extreme cost-effectiveness of our engineered *E. coli* platform makes the application of highly concentrated crude extracts both practical and highly effective for agricultural use. Therefore, our results reflect the realistic operational potential of these two systems rather than their equimolar stoichiometric efficiency.

In conclusion, rather than serving as a definitive validation, this study provides a strong initial indication and proof-of-concept for targeting the *EpCHSA* gene in the management of the oil-tea tussock moth, *E. pseudoconspersa*. Furthermore, while utilizing the *E. coli* HT115 expression system offers a highly promising and cost-effective platform to mitigate the economic constraints of RNAi applications, we acknowledge that its efficacy is not yet fully established for immediate large-scale field use. The elucidation of crucial dose–response dynamics and temporal efficacy profiles provides valuable foundational laboratory data; however, to transition these findings into real-world biopesticide strategies, future studies must subject this target to more vigorous testing processes, including comprehensive field trials and environmental stability evaluations.

## Data Availability

The original contributions presented in this study are included in the article/Supplementary Materials. Further inquiries can be directed to the corresponding authors.

## Supplementary Materials

The following supporting information can be downloaded at https://www.mdpi.com/article/10.3390/insects17050453/s1, Table S1: List of Primers of *EpCHSA* gene for PCR; Table S2: List of The sequence information of *EpCHSA* used in phylogenetic analysis; Figure S1: Prediction of transmembrane domains and phylogenetic tree of *EpCHSA*.

## References

[1] Chen J., Tang H., Li J.X., Cheng J., Cao B.N. (2024). Major pests and diseases of *Camellia oleifera* and their control measures. J. Henan For. Sci. Technol..

[2] Wei C.Y., Ma Y.L. (2011). Risk assessment and control technologies for the oil-tea tussock moth (*Euproctis pseudoconspersa*). J. Jiangsu Agric. Sci..

[3] Liu X.M., Shan Y. (2020). Species, occurrence, risk groups, and control measures of major insect pests on *Camellia oleifera* in Guangxi region. J. Spec. Econ. Plants Anim..

[4] Christiaens O., Niu J.Z., Taning C.N.T. (2020). RNAi in insects: A revolution in fundamental research and pest control applications. Insects.

[5] Liu C.X., Ning X.Y., Zhao P., Wei Z.H., Xie Y.Q., Zhu L., Li Z., Liu X.X. (2026). Nanocarrier-mediated RNAi targeting glutamate decarboxylase synergizes with neonicotinoids for aphid control. Chem. Eng. J..

[6] Zhu K.Y., Merzendorfer H., Zhang W.Q., Zhang J.Z., Muthukrishnan S. (2016). Biosynthesis, turnover, and functions of Chitin in Insects. Annu. Rev. Entomol..

[7] Merzendorfer H. (2006). Insect chitin synthases: A review. J. Comp. Physiol. B-Biochem. Syst. Environ. Physiol..

[8] Ye W.L., Li T., Weng M.Q., Guo W.C., Xin F.Y., Yu W., Wu S.Q., Guo Y.J. (2025). RNAi-mediated silencing of chitin synthase 1 (chs1) disrupts molting and growth in *Monochamus alternatus*. Forests.

[9] Cohen E. (2001). Chitin synthesis and inhibition: A revisit. Pest Manag. Sci..

[10] Zhong X., Yu X.D., Zhang J.H., Xu J.J., Qin M.C., Cao M.X., Francis F., Xia L.Q. (2025). RNAi technologies for insect control in crop protection. Crop J..

[11] Mon H., Kobayashi I., Ohkubo S., Tomita S., Lee J.M., Sezutsu H., Tamura T., Kusakabe T. (2012). Effective RNA interference in cultured silkworm cells mediated by overexpression of *Caenorhabditis elegans* SID-1. RNA Biol..

[12] Walski T., De Schutter K., Cappelle K., Van Damme E.J.M., Smagghe G. (2017). Distribution of Glycan Motifs at the Surface of Midgut Cells in the Cotton Leafworm (*Spodoptera littoralis*) Demonstrated by Lectin Binding. Front. Physiol..

[13] Yanagihashi Y., Usui T., Izumi Y., Yonemura S., Sumida M., Tsukita S., Uemura T., Furuse M. (2012). Snakeskin, a membrane protein associated with smooth septate junctions, is required for intestinal barrier function in *Drosophila*. J. Cell Sci..

[14] Xu Q.X. (2024). Screening and Functional Studies of Intestinal Insecticidal Target Genes Through RNAi in *Tribolium castaneum*. Master’s Thesis.

[15] Yang Y.Q., Liang Y.J., Zhi J.R., Li D.Y., Li C. (2024). Regulatory effect of trehalose metabolism on chitin synthesis in *Spodoptera frugiperda* (J. E. Smith) (Lepidoptera: Noctuidae) as determined using RNAi. J. Asia-Pac. Entomol..

[16] Timmons L., Court D.L., Fire A. (2001). Ingestion of bacterially expressed dsRNAs can produce specific and potent genetic interference in Caenorhabditis elegans. Gene.

[17] Livak K.J., Schmittgen T.D. (2001). Analysis of relative gene expression data using real-time quantitative PCR and the 2^−ΔΔCT^ method. Methods.

[18] Miao Z.G., Hong N., Liu D.Y., Xu P.J., Deng Q., Yan F.F., Yu J.M., Li M.Y., Liu S. (2025). Molecular characterization and functional analysis of the chitin synthase 1 gene from *Myzus persica*. Plant Prot..

[19] Du M.Y. (2020). Cloning and Functional Study of Chitin Synthase 2 Gene in Rusty Grain Beetle (*Cryptolestes ferrugineus*). Master’s Thesis.

[20] Zhang J.Z., Liu X.J., Zhang J.Q., Li D.Q., Sun Y., Guo Y.P., Ma E.B., Zhu K.Y. (2010). Silencing of two alternative splicing-derived mRNA variants of chitin synthase 1 gene by RNAi is lethal to the oriental migratory locust, *Locusta migratoria manilensis* (Meyen). Insect Biochem. Mol. Biol..

[21] Li J., Du J., Li S.W. (2016). Silence chitin synthase A gene in the rice leaf folder, *Cnaphalocrocis medinalis* (Lepidoptera: Pyralidae), by RNA interference. J. Mt. Agric. Biol..

[22] Jiang L.H., Mu L.L., Jin L., Anjum A.A., Li G.Q. (2021). RNAi for chitin synthase 1 rather than 2 causes growth delay and molting defect in *Henosepilachna vigintioctopunctata*. Pestic. Biochem. Physiol..

[23] Zeng B., Chen F.R., Sun H., Liu Y., Wu S.F., Bass C., Gao C.F. (2023). Molecular and functional analysis of chitin synthase genes in *Chilo suppressalis* (Lepidoptera: Crambidae). Insect Sci..

[24] Long G.J., Liu X.Z., Guo H., Zhang M.Q., Gong L.L., Ma Y.F., Dewer Y., Mo W.J., Ding L.W., Wang Q. (2024). Oral-based nanoparticle-wrapped dsRNA delivery system: A promising approach for controlling an urban pest, Blattella germanica. J. Pest Sci..

[25] Liu X.J., Zhang H.H., Li S., Zhu K.Y., Ma E.B., Zhang J.Z. (2012). Characterization of a midgut-specific chitin synthase gene (LmCHS2) responsible for biosynthesis of chitin of peritrophic matrix in *Locusta migratoria*. Insect Biochem. Mol. Biol..

[26] Ye C., Jiang Y.D., An X., Yang L., Shang F., Niu J.Z., Wang J.J. (2019). Effects of RNAi-based silencing of chitin synthase gene on moulting and fecundity in pea aphids (*Acyrthosiphon pisum*). Sci. Rep..

[27] Rana S., Rajurkar A.B., Kumar K.K., Mohankumar S. (2020). Comparative analysis of chitin synthasea dsrna mediated rna interference for management of crop pests of different families of Lepidoptera. Front. Plant Sci..

[28] Prince B.C., Chan K.L., Rueckert C. (2023). Elucidating the role of dsRNA sensing and Toll6 in antiviral responses of *Culex quinquefasciatus* cells. Front. Cell. Infect. Microbiol..

[29] Nishide Y., Kageyama D., Yokoi K., Jouraku A., Tanaka H., Futahashi R., Fukatsu T. (2019). Functional crosstalk across IMD and Toll pathways: Insight into the evolution of incomplete immune cascades. Proc. R. Soc. B-Biol. Sci..

[30] Yang F. (2023). Study on the Adaptability of *Spodoptera frugiperda* to Three Host Plants and the Function of Detoxification Genes. Master’s Thesis.

[31] Jian Q., Zong F.L. (2025). Brief analysis of environmental risk assessment considerations for topically applied dsRNA-based pesticides. Pestic. Sci. Adm..

[32] Edwards C.H., Baird J., Zinser E., Woods D.J., Shaw S., Campbell E.M., Bowman A.S. (2018). RNA interference in the cat flea, *Ctenocephalides felis*: Approaches for sustained gene knockdown and evidence of involvement of Dicer-2 and Argonaute2. Int. J. Parasitol..

[33] Terenius O., Papanicolaou A., Garbutt J.S., Eleftherianos I., Huvenne H., Kanginakudru S., Albrechtsen M., An C.J., Aymeric J.L., Barthel A. (2011). RNA interference in Lepidoptera: An overview of successful and unsuccessful studies and implications for experimental design. J. Insect Physiol..

[34] Zhang X., Fan Z.Z., Wang Q.H., Kong X.B., Liu F., Fang J.X., Zhang S.F., Zhang Z. (2022). RNAi efficiency through dsRNA injection is enhanced by knockdown of dsRNA nucleases in the fall webworm, *Hyphantria cunea* (Lepidoptera: Arctiidae). Int. J. Mol. Sci..

[35] Kolge H., Kadam K., Ghormade V. (2023). Chitosan nanocarriers mediated dsRNA delivery in gene silencing for *Helicoverpa armigera* biocontrol. Pestic. Biochem. Physiol..

[36] Kennedy S., Wang D., Ruvkun G. (2004). A conserved siRNA-degrading RNase negatively regulates RNA interference in *C. elegans*. Nature.

[37] Guan R.B., Li H.C., Fan Y.J., Hu S.R., Christiaens O., Smagghe G., Miao X.X. (2018). A nuclease specific to Lepidopteran insects suppresses RNAi. J. Biol. Chem..

[38] Chao Z.J., Ma Z.Z., Zhang Y.H., Yan S., Shen J. (2023). Establishment of star polycation-based RNA interference system in all developmental stages of fall armyworm *Spodoptera frugiperda*. Entomol. Gen..

[39] Taracena M., Hunt C., Pennington P., Andrew D., Jacobs-Lorena M., Dotson E., Wells M. (2022). Effective Oral RNA Interference (RNAi) Administration to Adult Anopheles gambiae Mosquitoes. Jove-J. Vis. Exp..

[40] Ganbaatar O., Cao B.D., Zhang Y.A., Bao D.R., Bao W.H., Wuriyanghan H. (2017). Knockdown of *Mythimna separata* chitinase genes via bacterial expression and oral delivery of RNAi effectors. BMC Biotechnol..

[41] Israni B., Rajam M.V. (2017). Silencing of ecdysone receptor, insect intestinal mucin and sericotropin genes by bacterially produced double-stranded RNA affects larval growth and development in *Plutella xylostella* and *Helicoverpa armigera*. Insect Mol. Biol..

[42] Vélez A.M., Fishilevich E. (2018). The mysteries of insect RNAi: A focus on dsRNA uptake and transport. Pestic. Biochem. Physiol..

[43] Nyström T. (2004). Stationary-phase physiology. Annu. Rev. Microbiol..

[44] Joga M.R., Zotti M.J., Smagghe G., Christiaens O. (2016). RNAi Efficiency, Systemic Properties, and Novel Delivery Methods for Pest Insect Control: What We Know So Far. Front. Physiol..

[45] Cagliari D., Dias N.P., Galdeano D.M., dos Santos E.A., Smagghe G., Zotti M.J. (2019). Management of Pest Insects and Plant Diseases by Non-Transformative RNAi. Front. Plant Sci..

[46] Cappelle K., de Oliveira C.F.R., Van Eynde B., Christiaens O., Smagghe G. (2016). The involvement of clathrin-mediated endocytosis and two Sid-1-like transmembrane proteins in doublestranded RNA uptake in the Colorado potato beetle midgut. Insect Mol. Biol..

[47] Christiaens O., Smagghe G. (2014). The challenge of RNAi-mediated control of hemipterans. Curr. Opin. Insect Sci..

[48] Fire A., Xu S.Q., Montgomery M.K., Kostas S.A., Driver S.E., Mello C.C. (1998). Potent and specific genetic interference by double-stranded RNA in *Caenorhabditis elegans*. Nature.

[49] da Rosa J., Viana A.J.C., Ferreira F.R.A., Koltun A., Mertz-Henning L.M., Marin S.R.R., Rech E.L., Nepomuceno A.L. (2024). Optimizing dsRNA engineering strategies and production in *E. coli* HT115 (DE3). J. Ind. Microbiol. Biotechnol..

[50] Zotti M., dos Santos E.A., Cagliari D., Christiaens O., Taning C.N.T., Smagghe G. (2018). RNA interference technology in crop protection against arthropod pests, pathogens and nematodes. Pest Manag. Sci..

[51] Nwokeoji A.O., Nwokeoji E.A., Chou T., Togola A. (2022). A novel sustainable platform for scaled manufacturing of double-stranded RNA biopesticides. Bioresour. Bioprocess..

