# Supplementary material for: Comparison of T7 In Vitro Transcription and E. coli Expression Systems for RNAi-Based Control of Euproctis pseudoconspersa by Targeting EpCHSA
Source: Insects. 2026 Apr 24;17(5):453. doi: 10.3390/insects17050453 (PMC13207690; doi:10.3390/insects17050453)
Supplement: Supplementary file 1 [file insects-17-00453-s001.zip › insects-4176521-supplementary.pdf]

Table S1 Primers of *EpCHSA* gene for PCR

Primers	Sequences（5′ -3′）
EpCHSA-F01	ACAGTACAAGAAACGAAAGG
EpCHSA-R01	GACTTCAAGAACTCCATCAT
EpCHSA-F02	TGCGCTACTATGTGGCA
EpCHSA-R02	TGCAGCCAATGACCAAT
EpCHSA-F11	CGAAACAGGACAGTGGGT
EpCHSA-R11	TATGGCTGGGATAATGCA
EpCHSA-F31	CGCCACAAGTTCTTCACC
EpCHSA-R31	TATCGGTAGTTCCATTAGACG
EGFP-F	TGCCCATCCTGGTCGAGCT
EGFP-R	TGCTTGTCGGCCATGATAT
T7F11	GGATCCTAATACGACTCACTATAGGACGAAACAGGACAGTGGG T
T7R11	GGATCCTAATACGACTCACTATAGGGTATGGCTGGGATAATGCA
T7F31	GGATCCTAATACGACTCACTATAGGACGCCACAAGTTCTTCACC
T7R31	GGATCCTAATACGACTCACTATAGGCTATCGGTAGTTCCATTAGA CG
EpCHSA -S F11	CGAGCTCGCGAAACAGGACAGTGGGT
EpCHSA-H R11	TAAGCTTGTATGGCTGGGATAATGCA
EpCHSA-S F31	CGAGCTCGCGCCACAAGTTCTTCACC
EpCHSA-H R31	TAAGCTTGTATCGGTAGTTCCATTAGACG
L4440-F	ACTATAGGGAGACCGGCAGAT
L4440-R	GGGAAGAAAGCGAAAGGAGC
EGFP-S F	CGAGCTCGTGCCCATCCTGGTCGAGCT
EGFP-H R	TAAGCTTGTGCTTGTCGGCCATGATAT
EpCHSA-QF1	CGATAGTCGCTCAAGTCA
EpCHSA-QR1	CGTGTAACAAAGTTCTCCC
β-actin-F1	GTTACTCATTCACCACCACCGCTG
β-actin-R1	GGATACCGCAAGATTCCATACCCA

Table S2 The sequence information of *EpCHSA* used in phylogenetic analysis

Gene Sequence	GenBank Accession No.	Species
MsCHSB	AY821560.1	*Manduca sexta*
MsCHS	AY062175	*Manduca sexta*
CmCHSB	KP000844.1	*Cnaphalocrocis medinalis*
SeCHSA	KT932387.1	*Spodoptera exigua*
HcCHSA	ON624330.1	*Hyphantria cunea*
AiCHSA	OL826753.1	*Agrotis ipsilon*
AiCHSA	OL826752.1	*Agrotis ipsilon*
HaCHSA	KP939100.1	*Helicoverpa armigera*
HaCHSAa	KP728469.1	*Helicoverpa armigera*
SfCHSA	OR669301.1	*Spodoptera frugiperda*
MbCHSA	GQ281761.2	*Mamestra brassicae*
MsCHSA	KT948989.1	*Mythimna separata*
MsCHS	AY062175.2	*Mythimna separata*
BmHAS	NM_001258362.1	*Bombyx mori*
ZcCHSB	XM_038355663.1	*Zerene cesonia*
CsCHSA	MT409245.1	*Chilo suppressalis*
CpCHSA	MN047452.3	*Chilo partellus*

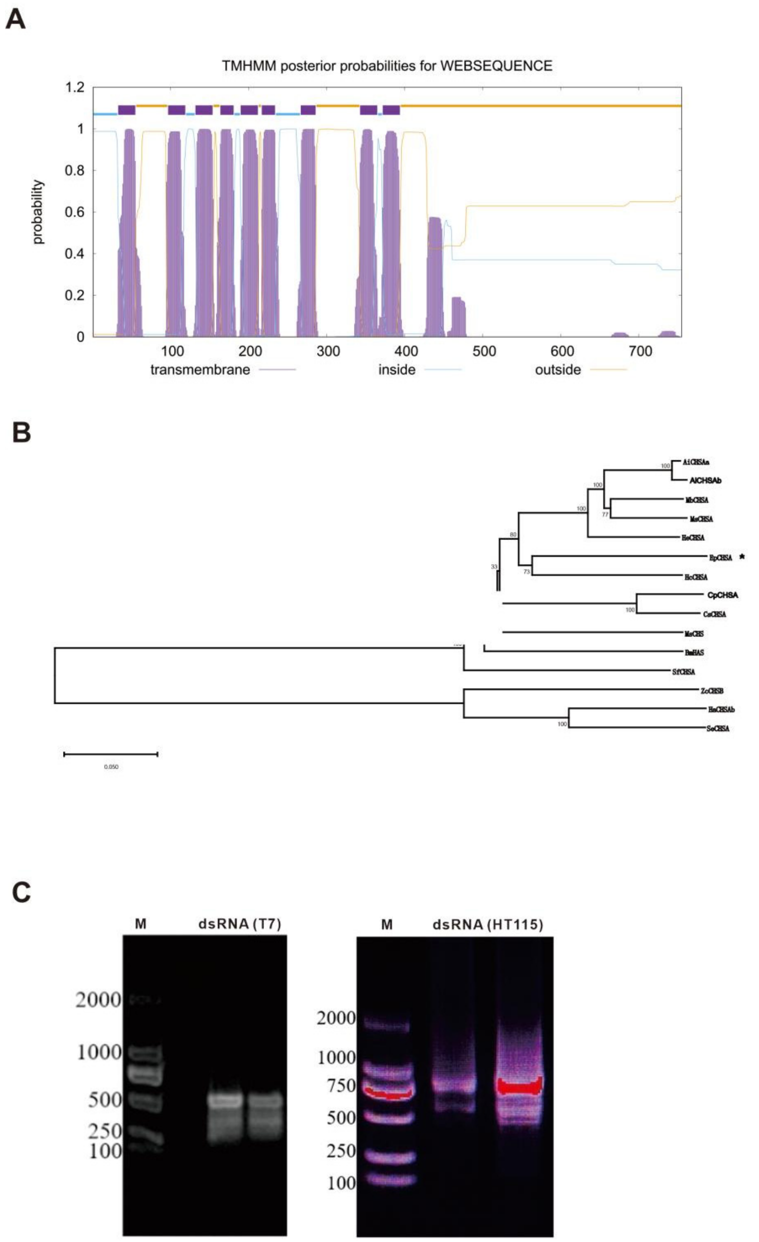

Figure S1. Prediction of transmembrane domains (A), phylogenetic tree (B) of *EpCHSA* and agarose gel electrophoresis analysis of synthesized dsRNA (C). Lane M: DNA Marker; Lane dsRNA(T7): dsRNA synthesized via in vitro transcription; Lane dsRNA(HT115): Total RNA extracted from engineered *E. coli* HT115 expressing target dsRNA, showing the target band along with endogenous bacterial RNAs.
